# Supplementary material for: ShinyAIM: Shiny‐based application of interactive Manhattan plots for longitudinal genome‐wide association studies
Source: Plant Direct. 2018 Oct 24;2(10):e00091. doi: 10.1002/pld3.91 (PMC6508828; doi:10.1002/pld3.91)
Supplement: Supplementary file 1 [file PLD3-2-e00091-s001.pdf]

Dear Editorial Board,

We will like to submit the revised manuscript ID (# 2018-00190) entitled “ShinyAIM: Shiny-based Application of Interactive Manhattan Plots for Longitudinal Genome-Wide Association Studies”. We appreciate the editor and reviewers for the thoughtful comments and suggestions on this manuscript. We have revised the manuscript accordingly and provide a point-by-point response to all comments. Our responses are highlighted in bold.

Sincerely,

Waseem Hussain (corresponding author)
Postdoctoral Research Associate
Departments of Animal Science & Agronomy
University of Nebraska-Lincoln
Email: waseem.hussain@unl.edu
WWW: https://whussain2.github.io/

Response to Editor

1) As stated by reviewer 2, it would be useful to have more information on how to run the app locally (using the shiny::runGitHub function), to allow more flexibility in the use of the app (en potentially the analysis of large data files, as stated by reviewer 1).

   Thank you for your suggestion. Information on how to run the application locally by running the code "shiny::runGitHub("ShinyAIM", "whussain2")" within R or RStudio has been incorporated in current version of the manuscript. Please see the lines 30, and 73-78 in the revised manuscript for more details. The text has also been updated on the "Information tab" of the application and also in the main page of shiny repository on GitHub.

2) I would advise to give a doi to the current version of the code, such as a static version of the app is linked to the manuscript. See here for instructions: https://guides.github.com/activities/citable-code/

   The DOI number has been assigned to the current version of the code. The DOI number is https://doi.org/10.5281/zenodo.1422835. The DOI number has been incorporated in the main page of application repository on GitHub and also in the "Information tab" of the application.

3) Make the examples files easier to download. As of now, the link from the web app re-direct toward a Github page, to a direct download, which can be confusing for users not familiar with Github. Maybe use the download Button element from Shiny?

   Download button in the application has been embedded in the main tabs. The users can download the sample files by just clicking on the download button. This information has been updated in the application and also in the manuscript from line number 89-91.

<u>Response to Reviewer #1</u>

1) The app is having an upper-limit of SNPs that can be viewed. At the moment the GWAS data contains up to 10m of SNPs that can be mapped, and it is likely that the server might not keep up with such a huge dataset.

   We agree with the reviewer that there is an upper limit for markers that can be interactively visualized in our application hosted on the shiny server. The application hosted on server can easily handle 200-300k SNPs. However, for the dynamic visualization of huge data (e.g., having millions of SNPs) the application can be launched locally if high computational resources are available for the user. This information has been added in the revised manuscript in the lines 92-96.

2) The authors use (I assume) Arabidopsis, displaying 5 chromosomes, but do not mention whether the number of the chromosomes different from 5 can be used in the app.

   Please note that the sample files provided in the application are rice subset data for the 12 chromosomes. The application can handle any number of chromosomes by providing the chromosome column "chrom" in the input data file.

3) The authors only refer to the online tutorial for the input file format. I think although it is good to have this online repository, it would also be worthwhile to discuss roughly what information MUST be included (position, chromosome, p-value / LOD score) and which CANNOT (simply because they are not being dealt with by the app), such as Minor Allele Frequency.

   This information has been incorporated in the manuscript. Please see the lines 81-91 in the revised manuscript.

4) Data visualization tab is a bit odd for this manuscript, as the tool is meant to visualize the GWAS results, rather than the general trends observed in the population scored. The rationale behind this tab should be therefore presented more clearly.

   Thank you for your comment. Please note that this shiny application was developed to interactively visualize results obtained from longitudinal GWAS by combining phenotypes collected over multiple time points with genetic marker information. The Interactive Manhattan Plot, Manhattan Grid Plot, and Comparison of Associated Markers tabs aim to visualize the strength of longitudinal marker effects wheres the Phenotypic Data visualization tab visualizes the longitudinal curve of phenotype. Because the temporal

GWAS results may depend on phenotypic trajectories, we believe that the Data visualization tab can also be useful for users. We have highlighted this point in the lines 56-62 of the revised manuscript.

5) The authors should also stress that this tool is not performing GWAS by itself, as they compare it to many tools that actually DO GWAS, rather than only visualize GWAS results.
This has been added in the revised manuscript. Please see line numbers 83-84.

6) The app is missing the M&M section, where the authors should at least list the libraries that they were using for developing individual sections of the app and the main "graph types".
Please note that the Materials and methods have been discussed under the sub-heading 'Implementation' in the earlier version of manuscript. However, to make it clearer, heading 'Implementation' has been renamed to 'Methods' and also texts have been changed in the paragraph. Please see the lines 63-80 in the revised manuscript.

7) Maybe this is not an abbreviation, but I missed why the application is called ShinyAIM - is this an abbreviation?
We choose the name ShinyAIM so as to make it simple, easy to pronounce, read and remember. The reason we choose Shiny because the application is built in Shiny and 'AIM' as acronym which mean Application for Interactive Manhattan Plots.

8) in lines 38-41 "For example, the application of GWAS to responses to abiotic stress, such as drought, salinity and temperature tolerance, measured at temporal resolution may provide insights into the mechanisms underlying plant physiological processes measured through the duration of stress or development"
- First of all "temperature tolerance" is not "abiotic stress" but something acquired by the plant
- the application of GWAS itself does not provide insight, but rather study of the genes identified by GWAS and allelic variation therein
The term temperature tolerance has been rephrased with temperature stress. We believe the application of GWAS includes identifying candidate QTL regions or genes and investigate their potential functional roles. Please see the lines 37-42 in the revised manuscript.

9) lines 72-73 "The ShinyAIM application does not require any working knowledge of R and is intuitively operated by mouse clicks." - instead of "mouse clicks" I would suggest putting a "graphical user interface".

This change has been made. Please see the line 72-73 in the revised manuscript.

Response to Reviewer #2:

The manuscript "ShinyAIM: Shiny-based Application of Interactive Manhattan Plots for Longitudinal Genome-Wide Association Studies" by Waseem Hussain, Malachy Campbell, Harkamal Walia and Gota Morota presents an intuitive way to visualize GWAS data via Manhattan Plots. In particular its ability to comprehensively visualize multiple Manhattan plots in various ways makes it unique. This is the presentation of a novel tool, not a study. Therefore, I can't comment on the scientific aspects of it as there are none. I think the tool will be useful to some researchers. The web-interface works well. I haven't tested it locally in R.
The manuscript is written well and describes the tool in a concise and clear manner. The authors did a good job presenting this online tool.
These are my suggestions:

1) I noticed a spelling error in the web interface: "Phenotypic Data Visulaization"
This change has been made.

2) There are no good instructions how to run this locally from the downloaded github package. It would be good to have instructions for that.
The Instructions have been added in the lines 30, and 73-79 in the revised manuscript.

3) While not necessary, the utility of the web-interface for time series could be enhanced by implementation of a Bonferoni and/or FDR sliding scale for a threshold and implementing heat map visualization of markers (like Fig. 1 from Kwak IY et al. (2014) Genetics. PMID: 24931408).
Although the current application only lists -log10 p-values, we agree that the multiple testing correction feature is something that potential users might appreciate. We will seriously consider to add this feature in the near future to enhance the utility of this application.
